# An oral–gut microbial metabolite links *Fusobacterium nucleatum* to aggravated myocardial ischemia–reperfusion injury

**DOI:** 10.1080/19490976.2026.2662082

**Published:** 2026-04-25

**Authors:** Yiwen Li, Qian Xu, Mengmeng Zhu, Wenting Wang, Yanfei Liu, Hongjun Yang, Yue Liu

**Affiliations:** a Beijing Key Laboratory of Traditional Chinese Medicine Basic Research on Prevention and Treatment for Major Diseases, Experimental Research Center, China Academy of Chinese Medical Sciences, Beijing, China; b National Clinical Research Center for TCM Cardiology, Xiyuan Hospital of China Academy of Chinese Medical Sciences, Beijing, China; c Department of Traditional Chinese Medicine, Chinese Academy of Medical Sciences and Peking Union Medical College, Beijing, China; d The Second Department of Geriatrics, Xiyuan Hospital, China Academy of Chinese Medical Sciences, Beijing, China; e Key Laboratory of Disease and Syndrome Integration Prevention and Treatment of Vascular Aging, Xiyuan Hospital of China Academy of Chinese Medical Sciences, Beijing, China; f China Academy of Chinese Medical Sciences, Beijing, China

**Keywords:** myocardial ischemia–reperfusion injury, oral–gut axis, *Fusobacterium nucleatum*, imidazole propionate, autophagy

## Abstract

**Background:**

Myocardial ischemia–reperfusion injury (MIRI) remains a major complication. *Fusobacterium nucleatum* (*F. nucleatum*), an oral pathobiont associated with cardiometabolic disease, may influence host physiology by reshaping gut microbial function through an oral–gut axis. Whether such microbial interactions contribute to MIRI remains unclear.

**Methods:**

An oral *F. nucleatum* gavage mouse model and cohorts were established to investigate the effect of oral *F. nucleatum* on MIRI, gut microbial histidine metabolism including imidazole propionate (ImP) production, the association of ImP with coronary heart disease (CHD), and its microbial sources. MIRI was induced with or without antibiotic-mediated microbiota depletion and/or ImP administration, and p62 dependence was examined by knockdown approaches *in vitro* and *in vivo*. Plasma metabolites, cardiac injury, ultrastructure, and p62/mTOR signaling were assessed.

**Results:**

*F. nucleatum* aggravated MIRI despite the absence of persistent colonic colonization. Instead, *F. nucleatum* altered gut microbial composition, including *Lactobacillus* abundance, and was associated with elevated circulating ImP. Antibiotic-mediated microbiota depletion reduced ImP and attenuated myocardial injury. Plasma ImP was elevated in patients with CHD, and ImP-producing capacity was supported primarily by gut microbiota urocanate reductase (UAR)-associated functions. In H9c2 cells, ImP exacerbated hypoxia/reoxygenation injury, and increased the autophagy adaptor p62 together with downstream mTOR/S6K1 signaling. p62 knockdown attenuated the mTOR/S6K1 response and injury-associated changes, whereas IRS1 suppression persisted.

**Conclusions:**

*F. nucleatum* reshapes gut microbial metabolism, thereby amplifying MIRI via ImP. ImP emerges as a functional mediator linking oral dysbiosis to MIRI, and reducing microbiota-derived ImP may represent a more mechanistically grounded strategy to mitigate MIRI.

## Highlights

- Oral pathobionts can aggravate MIRI by reshaping gut microbial metabolism via an oral–gut axis.
- *Fusobacterium nucleatum* is associated with gut urocanate reductase-positive microbial function, elevated circulating imidazole propionate (ImP).
- ImP activates p62/mTOR/S6K1 and reduces IRS1, with autophagy changes.

## Graphical abstract

**Figure:**
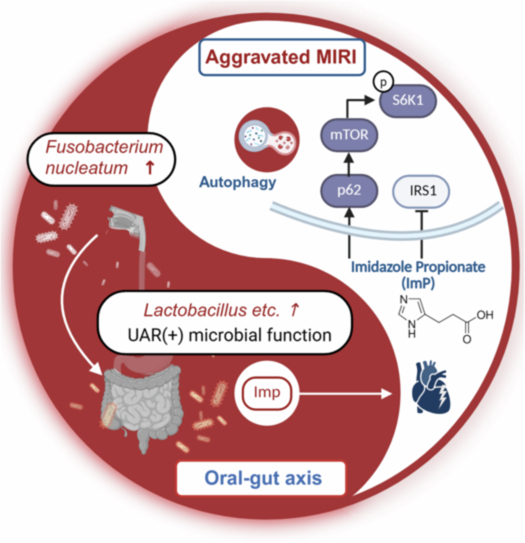

## Introduction

Coronary heart disease (CHD) remains a leading cause of death and disability worldwide.^1^ Although timely revascularization is the cornerstone of CHD management, myocardial ischemia-reperfusion injury (MIRI) continues to limit the net benefit of reperfusion therapy.^2^ Importantly, the severity of MIRI varies markedly among individuals. This heterogeneity is shaped not only by ischemic burden and procedural factors, but also by broader metabolic determinants.^3‐5^ Among these, microbiota have emerged as important modulators.^6^^,^^7^

Oral dysbiosis has emerged as an independent risk factor for adverse cardiovascular events.^8^ Among oral pathobionts, *Fusobacterium nucleatum* (*F. nucleatum*), a key periodontal pathogen, is consistently enriched in patients with CHD.^9‐12^ However, its pathogenic role in cardiovascular injury remains incompletely understood. A common explanation is that disruption of the oral or gut barrier allows microbial products or *F. nucleatum* itself to enter the circulation and aggravate injury.^13‐15^ However, this model does not fully account for several observations. *F. nucleatum* can worsen intestinal and extra-intestinal pathology^16^ even in models without overt barrier disruption, yet it does not consistently establish stable colonization in the healthy gut.^17^^,^^18^ These findings suggest that, beyond direct pathogenic effects, *F. nucleatum* may influence disease progression through interactions with microbial ecosystems.

An emerging alternative framework proposes that oral pathobionts can act as ecological drivers that reshape gut microbial composition and metabolic output, thereby affecting systemic disease through an oral–gut axis.^13^^,^^19^ This is consistent with our previous observations: oral enrichment of *F. nucleatum* was associated with altered gut microbial features, notably an increase in gut *Lactobacillus*, a metabolically active bacterial taxon, rather than enrichment of gut *F. nucleatum* itself.^20‐22^ These findings suggest that the oral–gut axis may provide an additional explanation for how oral pathogens influence cardiovascular injury,^19^^,^^23^^,^^24^ beyond the classical barrier-disruption model.

Based on these observations, we sought to determine whether an *F. nucleatum*-driven oral–gut microbial axis promotes cardiotoxic metabolite production primarily by remodeling gut microbial function. To address this question, we combined CHD clinical cohorts with complementary mouse and cell experiments to: (i) define oral *F. nucleatum*-associated oral–gut microbial interactions and their relationship to MIRI; (ii) identify key circulating metabolites, infer its microbial origin, and nominate candidate microbial contributors; and (iii) delineate the cardiac target and signaling pathway engaged by this metabolite using microbiota depletion, metabolite intervention, and gene-silencing approaches. Overall, our findings support an oral–gut axis in which oral *F. nucleatum* reshapes gut microbial metabolic function, elevates circulating imidazole propionate (ImP), and thereby aggravates MIRI while perturbing cardiac autophagy signaling.

## Methods

### Cohort information

Patients were recruited from the Department of Cardiology, Xiyuan Hospital of China Academy of Chinese Medical Sciences (Beijing, China). Based on the presence or absence of coronary heart disease (CHD), participants were categorized into a CHD group and a metabolically comparable control (MCC) group. Control participants were enrolled with simple randomization, and baseline analyses showed that the two groups were broadly comparable in age, sex, body mass index (BMI), and glycemic status. The study protocol was approved by the Ethics Committee of Xiyuan Hospital, China Academy of Chinese Medical Sciences (Approval No. 2024XLA036-2) and registered at the Chinese Clinical Trial Registry (ChiCTR2400084255). Written informed consent was obtained from all participants. Clinical information, including demographic characteristics, blood pressure, BMI, medical history, duration of diabetes, and antidiabetic medication use, was collected at enrollment. Fasting venous blood samples were obtained on the day of admission or the following morning for routine laboratory testing, including complete blood count, glycemic indices, lipid profile, liver and renal function markers, cardiac troponin, and *N*-terminal pro-B-type natriuretic peptide (NT-proBNP).

### Metagenomic analysis of clinical oral and gut microbiota

Metagenomic sequencing data (PRJCA024368) were used for source contribution analyzes of histidine ammonia-lyase (HAL) and urocanate reductase (UAR) within the oral and gut microbiomes of the clinical cohort.

Raw paired-end metagenomic sequencing reads were subjected to quality control and adapter trimming using Trim Galore v0.5.0 and Cutadapt v1.11. Host-derived reads were removed by aligning the filtered reads to the human reference genome (hg38) using Bowtie2 v2.1.0. Taxonomic profiling was performed using MetaPhlAn3 based on the ChocoPhlAn database to obtain microbial composition and relative abundance at different taxonomic levels. Functional profiling was conducted using HUMANN3 to generate gene family and MetaCyc pathway abundance tables.

Downstream analyzes were mainly based on KO abundance profiles. Target functional features were extracted and compared among groups according to the sample metadata. Data visualization and statistical analyses were performed in R, mainly using the packages ggplot2, ggpubr, and related packages. In addition, species-level contribution to specific KO functions was assessed by integrating microbial contribution results with phylogenetic information, and visualized using the ape, ggtree, dplyr, and tidyr packages in R.

### Animal experiments

Male C57BL/6J wild-type mice (6 weeks of age; 16–20 g) were purchased from SPF Biotechnology Co., Ltd. (Beijing, China; production license SCXK [Beijing] 2024-0001). Mice were housed in a specific pathogen-free (SPF) facility under controlled conditions with ad libitum access to sterilized standard chow and water. All animal procedures were conducted in accordance with relevant ethical guidelines and animal welfare regulations.

To induce microbiota dysbiosis, mice were administered *F. nucleatum* (ATCC 25586) or *Escherichia coli* DH5α (*E. coli,* BNCC 353719) suspension by oral gavage for 6 weeks. During the first 2 weeks, mice received 200 μL of *F. nucleatum* suspension every other day, containing 1 × 10^9^ colony-forming units (CFU) per gavage; during the subsequent 4 weeks, gavage was performed once weekly using the same dose.

All surgical procedures were performed within 1–4 h after the onset of the light cycle. Mice were anesthetized with isoflurane and placed in the supine position on a small-animal operating table with the limbs secured. The left anterior descending (LAD) coronary artery was identified and ligated with a sterile 7–0 silk suture using a slipknot. Successful ischemia was confirmed by blanching of the distal myocardium together with electrocardiographic changes, including ST-segment elevation and/or pathological T-wave changes. After 30 min of ischemia, the chest was reopened, and the ligature was released to allow reperfusion. Sham-operated mice underwent the same anesthesia and thoracotomy procedures with cardiac exposure, but without LAD ligation. Hearts and plasma samples were collected 24 h after reperfusion.

To generate antibiotic-treated pseudo-germ-free mice, animals received a broad-spectrum antibiotic cocktail by oral gavage once daily for 6 weeks (200 μL per dose). The cocktail contained ampicillin (1 g/L), neomycin sulfate (1 g/L), metronidazole (1 g/L), and vancomycin (0.5 g/L) dissolved in autoclaved sterile water.

For *in vivo* p62 knockdown, adeno-associated virus (AAV) was administered via tail-vein injection. Viral stocks were diluted in phosphate-buffered saline (PBS) to the desired concentration according to the manufacturer's instructions. Each mouse received 100 μL of virus at a dose of 3 × 10^11^ genome copies (GC)/mL. To ensure stable AAV-mediated p62 silencing, AAV was injected 4 weeks prior to model induction.

### Cell lines and culture conditions

H9c2 rat cardiomyoblasts were obtained from Wuhan YUANSHENG Original Primary Biomedicine Technology Co., Ltd. (catalog no. r037). Cells were cultured in Dulbecco's modified Eagle's medium (DMEM) supplemented with 10% fetal bovine serum (FBS) and 1% penicillin–streptomycin under standard conditions.

Hypoxia/reoxygenation (H/R) was used to mimic ischemia–reperfusion injury *in vitro*. For hypoxia, cells were incubated in glucose-free DMEM pre-equilibrated with a gas mixture of 95% N₂ and 5% CO₂ and maintained under hypoxic conditions for 6 h. Reoxygenation was initiated by replacing the medium with regular DMEM and incubating cells under normoxic conditions for an additional 2 h.

ImP was tested in H9c2 cells across a concentration range of 100–800 nM. Based on a concentration-response viability assessment, a stable and reproducible reduction in viability was observed at 400–600 nM; therefore, 500 nM was selected as the standard ImP concentration for subsequent experiments.

For transient gene silencing, H9c2 cells in the logarithmic growth phase were seeded and transfected at approximately 70–80% confluence with p62-specific siRNA or negative control siRNA. Transfection complexes were prepared according to the manufacturer's instructions and added to cells for incubation. After transfection, cells were maintained in culture and subsequently subjected to H/R and downstream assays. All oligonucleotides were synthesized by GenePharma (Shanghai, China). The p62 siRNA sequences were as follows: sense, GCUGCUGUCCGUAGAAAUUTT; antisense, AAUUUCUACGGACAGCAGCTT.

### Untargeted metabolomics of colonic contents

Untargeted metabolomic analysis of mouse colonic contents was performed using UHPLC–QE–MS to characterize microbiota-associated metabolite profiles and pathway alterations. Analyses were conducted by Metware Biotechnology Co., Ltd. (Wuhan, China). 20 mg of each sample was extracted with 400 μL of 70% methanol/water containing internal standards, followed by vortexing, ice-bath sonication, low-temperature incubation, and centrifugation. The resulting supernatants were subjected to UHPLC–MS analysis using a Waters ACQUITY Premier HSS T3 column (1.8 μm, 2.1 mm × 100 mm) with 0.1% formic acid in water (A) and acetonitrile (B) as the mobile phases. Data were acquired in both positive and negative electrospray ionization modes. Raw data were converted to mzML format and processed with XCMS for peak extraction, alignment, and retention time correction. Metabolite annotation was performed using an in-house database together with public and predicted libraries. Only metabolites with an integrated identification score > 0.5 and QC CV < 0.3 were retained for downstream analysis. Statistical analyses were performed as described in the Statistical Analysis section.

### Targeted metabolomics of plasma

Targeted metabolomic analysis was performed to quantify imidazole propionate (ImP) and urocanate in plasma samples using UPLC–MS/MS. Analyzes were conducted by Yitong Qijun Technology Co., Ltd. (Beijing, China). Briefly, 100 μL of plasma was extracted with 300 μL methanol, followed by vortexing, low-temperature sonication, incubation, and centrifugation, and the supernatants were collected for analysis. Chromatographic separation was performed on a PREMIER BEH Z–HILIC column (1.7 μm, 2.1 mm × 100 mm) using ammonium formate/ammonia-containing aqueous and acetonitrile-based mobile phases. Mass spectrometric detection was carried out with an electrospray ionization source in multiple reaction monitoring (MRM) mode. Peak integration was performed using MultiQuant software, and ImP and urocanate concentrations were calculated using standard curves.

### Cell counting kit-8 (CCK-8) assay

Cell viability of H9c2 cells was assessed using a Cell Counting Kit-8 (CCK-8) assay according to the manufacturer's instructions (Solarbio, Beijing, China; Cat. No. CA1211). H9c2 cells were seeded in 96-well plates and treated as indicated. After treatment, the medium was replaced and CCK-8 reagent was added, followed by incubation. Absorbance at 450 nm was measured using a microplate reader, and optical density (OD) values were used to quantify cell viability.

### Quantitative PCR (qPCR) for microbial DNA

Total DNA was extracted from mouse oral swabs and fecal samples using commercial kits (TIANGEN, Beijing, China; DP438; and Omega BioTek, Norcross, GA, USA; D5625). DNA concentration and purity were determined using a microvolume spectrophotometer (Merinton, China; SMA4000). SYBR Green-based quantitative PCR (qPCR) was performed to determine the relative abundance of *F. nucleatum* and *Lactobacillus* using an ABI 7500 real-time PCR system (Applied Biosystems, USA). Primer sequences are provided in Table 1.

**Table 1.** Primer sequences for qPCR.

Target organism	Primer (5′→3′)	Sequence (5′→3′)
*Lactobacillus*	Forward	GAGGCAGCAGTAGGGAATCTTC
*Lactobacillus*	Reverse	GGCCAGTTACTACCTCTATCCTTCTTC
*Fusobacterium nucleatum*	Forward	CAACCATTACTTTAACTCTACCATGTTCA
*Fusobacterium nucleatum*	Reverse	GTTGACTTTACAGAAGGAGATTATGTAAAAATC

### Transthoracic echocardiography

Transthoracic echocardiography was performed under 1.0–1.5% isoflurane anesthesia. Cardiac function was assessed using a Vevo 2100 high-resolution small-animal ultrasound system (VisualSonics). Parasternal long-axis views were acquired, and M-mode images were recorded at the level of the papillary muscles. Left ventricular end-diastolic and end-systolic internal diameters, as well as anterior and posterior wall thicknesses, were measured. Left ventricular ejection fraction (EF) and fractional shortening (FS) were calculated accordingly.

### Enzyme-linked immunosorbent assay (ELISA)

ELISA was performed according to the manufacturers' protocols. Mouse ELISA kits for HAL (MM-4777M2), UAR (MM-48469M2), insulin (INS; MM-0579M2), cardiac troponin I (cTnI; MM-46632M2), lactate dehydrogenase (LDH; MM-43732M2), interleukin-1β (IL-1β; MM-00404M2), interleukin-10 (IL-10; MM-0176M2), and tumor necrosis factor-α (TNF-α; MM-01332M2) were purchased from Jiangsu Enzyme Immunoassay Industry Co., Ltd. (Jiangsu, China). Levels of IL-1β (KE20021), IL-10 (KE20022), and TNF-α (KE20018) in cell culture supernatants were measured using ELISA kits from Proteintech (Wuhan, China).

### Hematoxylin and eosin (HE) staining

Heart, colon, and oral tissues were fixed in 4% paraformaldehyde for 24 h. Oral tissues were decalcified in EDTA solution. Samples were then processed by graded ethanol dehydration, xylene clearing, and paraffin embedding. Paraffin sections (4–5 μm) were deparaffinized, rehydrated, and stained with hematoxylin and eosin (HE). After dehydration and mounting, tissue morphology was examined under a light microscope.

### Periodic acid–Schiff (PAS) staining

Colon tissues were fixed, dehydrated, cleared, and paraffin-embedded using standard procedures. Paraffin sections (4 μm) were deparaffinized and rehydrated, followed by periodic acid–Schiff (PAS) staining. Sections were oxidized with periodic acid, developed with Schiff reagent, counterstained with hematoxylin, dehydrated, cleared, and mounted with neutral resin. Stained sections were examined under a light microscope.

### TUNEL staining

Paraffin-embedded heart sections were processed as described for H&E staining. After deparaffinization and rehydration, sections were washed with PBS and treated with 3% hydrogen peroxide at room temperature to block endogenous peroxidase activity. Sections were then digested with proteinase K to enhance tissue permeability. TUNEL staining was performed using a commercial kit (Servicebio, Wuhan, China; Cat. No. G1501) according to the manufacturer's instructions. Sections were incubated for terminal deoxynucleotidyl transferase (TdT) labeling at 37 °C in the dark. After washing, nuclei were counterstained with DAPI. Autofluorescence quenching was performed prior to mounting with an anti-fade mounting medium. Images were acquired using a fluorescence microscope.

### Tissue immunofluorescence (IF)

Paraffin sections were deparaffinized and rehydrated, followed by antigen retrieval and permeabilization. Sections were blocked with 3% bovine serum albumin (BSA) at room temperature and incubated overnight at 4 °C with primary antibodies against p62 (1:200, Abcam) and mTOR (1:150, Cell Signaling Technology). After PBS washes, sections were incubated with fluorescence-conjugated secondary antibodies at room temperature in the dark. Nuclei were counterstained with DAPI, and autofluorescence quenching was performed before mounting with an anti-fade medium. Images were acquired using a fluorescence microscope or a digital slide scanning system.

### Cellular immunofluorescence

H9c2 cells were seeded on coverslips. After treatment, cells were washed with PBS, permeabilized, and blocked with 3% BSA at room temperature to reduce nonspecific binding. Cells were incubated overnight at 4 °C with primary antibodies against mTOR (1:100, Abcam) and p62 (1:200, Abcam), followed by incubation with fluorescence-conjugated secondary antibodies. Nuclei were counterstained with DAPI. Coverslips were mounted and images were acquired using a fluorescence microscope or digital imaging/scanning system, followed by image analysis.

### Quantitative reverse transcription PCR (qRT–PCR)

Total RNA was extracted from heart tissues and H9c2 cells using TRIzol reagent (Invitrogen; 15596018CN). RNA concentration and purity were measured, and equal amounts of RNA were reverse-transcribed into cDNA using an M-MLV reverse transcription kit (Promega; M1701). Quantitative PCR was performed using a real-time PCR master mix (Qiagen) on an ABI 7500 Fast real-time PCR system. Primers were synthesized by Sangon Biotech (Shanghai, China). Primer sequences are provided in Table 2.

**Table 2.** Primer sequences for qRT–PCR (5′–3′).

Gene	Forward primer (5′→3′)	Reverse primer (5′→3′)
p62	CGACTGGACGCATTTGTCTT	GTCTAGAGAGCTTGGCCCTT
mTOR	AGAACCACATGCCACACAGT	CTTTGGCATTTGTGTCCATC
GAPDH	CAACTCCCTCAAGATTGTCAGCAA	GGCATGGACTGTGGTCATGA

### Western blotting

Western blotting was performed to assess protein expression in heart tissues and H9c2 cells. Total protein was extracted using RIPA lysis buffer supplemented with protease and phosphatase inhibitors. Protein concentration was determined using a bicinchoninic acid (BCA) assay. Equal amounts of protein were separated by SDS–PAGE and transferred to PVDF membranes. Membranes were blocked with 5% non-fat milk and incubated overnight at 4 °C with primary antibodies against mTOR, phospho-mTOR (p-mTOR), p62, phospho-p62 (p-p62), S6K1, phospho-S6K1 (p-S6K1), Beclin1, and LC3 (antibody details are provided in Table 3). After incubation with HRP-conjugated secondary antibodies, signals were visualized using enhanced chemiluminescence (ECL) and imaged. Band intensities were quantified using ImageJ software and normalized to GAPDH.

**Table 3.** Antibodies used for Western blotting.

Protein	Company	Catalog No.
mTOR	Abcam	ab32028
*p*-mTOR	Proteintech	67778-1-Ig
p62 (SQSTM1)	Abcam	ab109012
*p*-p62	Abcam	ab211324
S6K1	Abcam	ab32529
*p*-S6K1	Abcam	ab59208
Beclin 1	Proteintech	11306-1-AP
LC3B-II	Abcam	ab192890
ATG5	Proteintech	10181-2-AP
IRS1	CST	2382S
Bax	Proteintech	50599-2-Ig
Caspase-3	Abcam	ab13847
ZO-1	Invitrogen	61-7300
GAPDH	Abcam	ab181602

### Transmission electron microscopy (TEM)

Transmission electron microscopy was used to examine ultrastructural changes in heart tissues and cardiomyocytes. Samples were fixed in glutaraldehyde, postfixed in osmium tetroxide, dehydrated through a graded acetone series, and embedded in epoxy resin (Epon-812). Ultrathin sections (60–90 nm) were prepared, stained with uranyl acetate and lead citrate, and examined under a transmission electron microscope. Images were acquired for analysis.

### Statistical analysis

Statistical analyzes were performed using IBM SPSS Statistics version 23.0, and data visualization was conducted using GraphPad Prism version 10.0. Continuous variables are presented as mean ± SEM, and categorical variables are presented as numbers with percentages. Data were assessed for normality and homogeneity of variance. Categorical variables were analyzed using the chi-square test. For comparisons among multiple groups with one independent variable and homogeneity of variance, one-way analysis of variance (one-way ANOVA) was used. For nonnormally distributed data or data with unequal variances, the Kruskal–Wallis test was applied. For the four-group animal experiments, data were additionally analyzed by two-way ANOVA. Main effects of bacterial treatment and surgical condition, as well as their interaction, were evaluated. When an interaction effect was significant, post hoc multiple comparisons were performed to compare the effect. A two-sided *P* < 0.05 was considered statistically significant.

## Results

### Oral *F. nucleatum* remodels gut microbial composition and aggravates MIRI

**Figure 1.**
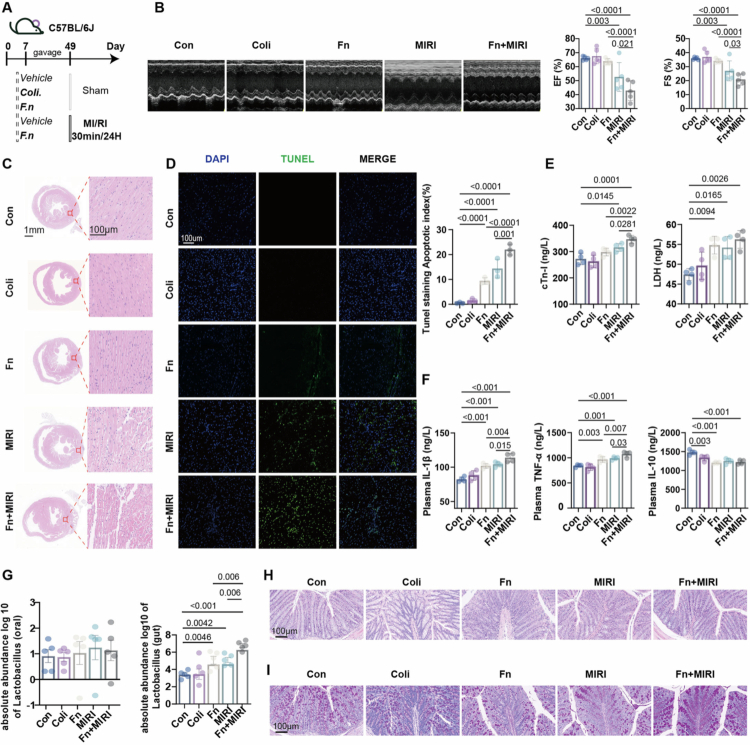
*F. nucleatum* mediates oral–gut axis and aggravates MIRI. (A) Schematic of the *in vivo* study design. Mice received oral gavage of *F. nucleatum* or *E. coli* and then underwent either sham surgery or LAD ligation/reperfusion. (B) Echocardiographic assessment of cardiac function and quantification of left ventricular ejection fraction (EF) and fractional shortening (FS) (*n* = 5). (C) Representative heart section staining (*n* = 3). (D) Representative TUNEL staining of heart sections (*n* = 3) and quantification of TUNEL-positive cells. (E) Plasma cTnI and LDH (*n* = 4). (F) Plasma IL-1β, TNF-*α*, and IL-10 (*n* = 4). (G) Abundance of *Lactobacillus* in oral and gut samples (*n* = 5). (H–I) Representative colon staining (*n* = 3).

Building on our prior clinical observations implicating an *F. nucleatum*-linked oral–gut axis, we tested whether oral *F. nucleatum* drives a species-specific oral–gut interaction and aggravates MIRI. Mice received oral gavage of *F. nucleatum*, with *E. coli* as a bacterial control, followed by sham or I/R surgery (Figure 1A). Compared with *E. coli*, oral *F. nucleatum* produced a markedly stronger injury phenotype: in I/R mice, *F. nucleatum* further reduced left ventricular ejection fraction and fractional shortening and increased myocardial apoptosis, accompanied by higher plasma cTnI and inflammatory cytokines (Figure 1B–F). *F. nucleatum* alone also increased myocardial inflammatory cytokines, apoptosis, and circulating LDH and cytokines, whereas *E. coli* gavage did not elicit comparable changes (Figure 1C–F). Two-way ANOVA showed significant interaction between *F. nucleatum* treatment and MIRI for systemic inflammatory cytokines (Figure S1), suggesting a stronger inflammatory effect of *F. nucleatum* in MIRI mice. Blood glucose levels and insulin resistance were increased after *F. nucleatum* exposure (Figure S2).

Microbiome profiling showed that oral *F. nucleatum* increased *Lactobacillus* abundance in the gut, whereas oral *Lactobacillus* remained unchanged (Figure 1G), supporting a cross-site oral–gut interaction rather than changes driven by within-oral community interactions. Consistent with this interpretation, *F. nucleatum* was not detectably maintained in the gut (Figure S3), arguing against sustained intestinal expansion as the primary driver of the gut community change. Despite the exacerbated cardiac phenotype, multiple assessments indicated that oral *F. nucleatum* exposure was not accompanied by overt disruption of oral or gut mucosal barrier integrity or local inflammatory activation under our experimental conditions (Figure 1H–I, Figure S4). This remodeling was exemplified by *Lactobacillus* enrichment and occurred without persistent colonic colonization.

### Oral *F. nucleatum* shifts gut microbiota function to increase imidazole propionate

**Figure 2.**
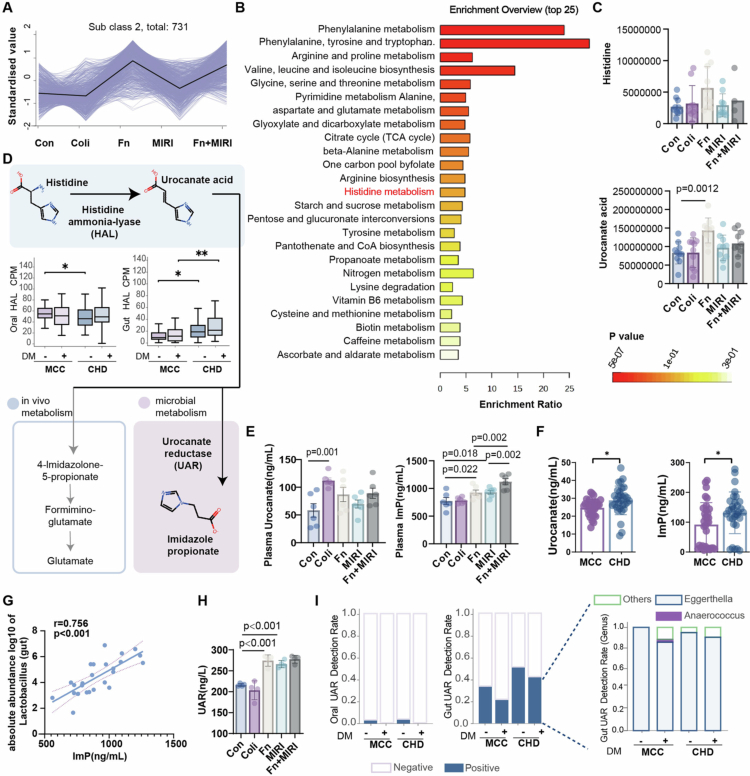
*F. nucleatum* remodels gut microbial function to elevate circulating ImP in mice and humans (A) k-means clustering identifying a metabolite module (Cluster 2). (B) Pathway enrichment analysis of metabolites from Cluster 2, (C) Colonic content histidine and urocanate levels. (D) Schematic of the HAL-mediated conversion of histidine to urocanate, together with comparison of HAL abundance (CPM) in oral and gut metagenomes from MCC and CHD participants. (E) Plasma urocanate and ImP levels (*n* = 6). (F) Plasma urocanate and ImP levels in CHD (*n* = 31) and MCC (*n* = 32). (G) Correlation between gut *Lactobacillus* abundance and ImP levels. (H) Plasma levels of UAR measured by ELISA (*n* = 4). (I) Relative abundance of UAR in oral and gut metagenomes. *P＜0.05, **P＜0.01.

Given that *F. nucleatum* aggravated MIRI without detectable gut colonization or overt barrier disruption, we examined whether it alters gut metabolic output to produce circulating mediators. Untargeted metabolomics of colonic contents revealed a pronounced shift in the gut metabolome after *F. nucleatum* exposure (Figure S5A). Differential feature filtering followed by k-means clustering and Venn analysis identified an *F. nucleatum*-responsive metabolite set (Figure 2A, Figure S5B, C, Table S1-2). KEGG enrichment of Cluster 2 highlighted histidine metabolism as the top altered pathway (Figure 2B), consistent with pathway enrichment based on differential metabolites between groups (Figure S5D). Within the histidine pathway, *F. nucleatum* selectively increased urocanate abundance in colonic contents (Figure 2C). Although other amino acid metabolites were also altered (Figure S6), *F. nucleatum* has a direct enzymatic link to histidine metabolism because it encodes HAL,^25^ which catalyzes the upstream histidine-to-urocanate step (Figures S7–S8).

Urocanate is the immediate substrate of UAR, the microbial enzyme responsible for the terminal conversion to ImP ^26‐28^ (Figure 2D), and can also serve as a terminal electron acceptor for UAR-positive bacteria under anaerobic conditions.^25^ This raised the possibility that increased urocanate might favor downstream UAR-dependent ImP production. Consistent with this model, targeted metabolomics showed that although plasma urocanate was not significantly altered in mice, *F. nucleatum* significantly increased circulating ImP (Figure 2E), supporting enhanced microbial conversion rather than simple precursor accumulation. Consistent with the mouse findings, plasma urocanate and ImP were also significantly elevated in a CHD cohort with a history of percutaneous coronary intervention (PCI) compared with the control group (MCC) (Figure 2F, Figures S9–S10, Table S4).

To infer the likely source of elevated ImP, we focused on the two metabolic steps underlying its production. *F. nucleatum* encodes the upstream HAL (Figure S7–8) step but lacks UAR^22^ (Table S3), the enzyme required for the terminal conversion to ImP. This supports a two-step model in which oral *F. nucleatum* may contribute to urocanate availability, whereas ImP-producing capacity resides in UAR-positive bacteria. In mice, gut *Lactobacillus* abundance was positively correlated with plasma ImP (Figure 2G), and *F. nucleatum* exposure increased UAR levels (Figure 2H). This pattern was further supported in the clinical cohort, in which UAR was more frequently detected in gut samples from patients with CHD, whereas oral UAR remained low (Figure 2I). Source-tracing analyzes indicate that *Lactobacillus* could be regarded as one candidate contributor^22^ (Figure 2I). Collectively, these findings support the gut microbiome, rather than the oral microbiome, as the likely proximal source of circulating ImP in CHD, and support a model in which oral *F. nucleatum* promotes a gut histidine-metabolic configuration favoring both urocanate accumulation and UAR-dependent ImP production.

### Gut microbiota depletion lowers ImP and mitigates MIRI

**Figure 3.**
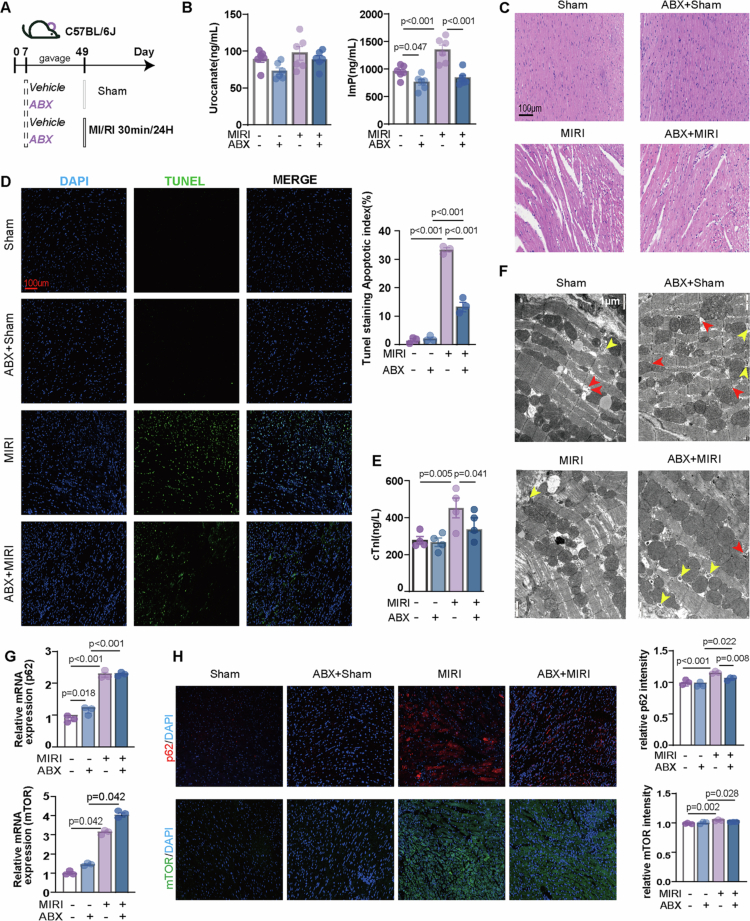
Microbiota depletion lowers circulating ImP and is associated with attenuated MIRI. (A) Schematic of the study design. Mice received antibiotics to deplete the gut microbiota and then underwent either sham or LAD ligation/reperfusion. (B) Plasma urocanate and ImP levels (*n* = 6). (C) Representative H&E staining of heart sections (*n* = 3). (D) Representative TUNEL staining of heart sections (*n* = 3). (E) Plasma cTnI levels (*n* = 4). (F) TEM of cardiac tissue (*n* = 3). Red arrows indicate autophagosomes, and yellow arrows indicate autolysosomes. (G) Cardiac p62 and mTOR mRNA levels (*n* = 3). (H) Immunofluorescence staining of cardiac p62 and mTOR (*n* = 3).

To assess the association of gut microbiota depletion with circulating ImP and MIRI severity, we generated pseudo-germ-free mice using broad-spectrum oral antibiotics (ABX) and then performed sham or I/R surgery (Figure 3A). ABX markedly reduced circulating ImP, whereas urocanate showed a downward trend without reaching statistical significance (Figure 3B). In the MIRI setting, ABX was associated with reduced myocardial injury, including decreased inflammatory cell infiltration and fewer TUNEL-positive cardiomyocytes, together with a blunted increase in plasma cTnI (Figure 3C–E). These protective associations were not accompanied by significant differences in systemic lipid indices or baseline inflammatory status (Figure S11).

At the ultrastructural level, ABX mitigated I/R-induced mitochondrial swelling and sarcomere disorganization. Compared with the MIRI group, the ABX + MIRI group displayed more recognizable autophagy-related structures, including autolysosome-like structures (Figure 3F), suggestive of partial restoration of autophagy-related ultrastructural features.

Given prior evidence linking ImP to p62-mTOR signaling,^22^^,^^29^ we next assessed myocardial p62/mTOR-related markers. I/R increased p62 and mTOR mRNA expression and enhanced myocardial p62 immunoreactivity (Figure 3G–H). Under MIRI conditions, ABX did not reverse the transcriptional upregulation (Figure 3G), but it significantly reduced myocardial p62 immunofluorescence intensity (Figure 3H), suggesting reduced tissue-level p62 accumulation despite persistent transcriptional activation. By contrast, immunoblotting of whole-heart lysates did not reveal a consistent change in total p62/mTOR abundance or phosphorylation ratios after ABX treatment (Figure S12), possibly because strong I/R-intrinsic activation and tissue heterogeneity^30^^,^^31^ masked more modest microbiota-dependent effects in bulk tissue analyzes.

### ImP exacerbates H/R injury by impairing autophagy in cardiomyocytes

**Figure 4.**
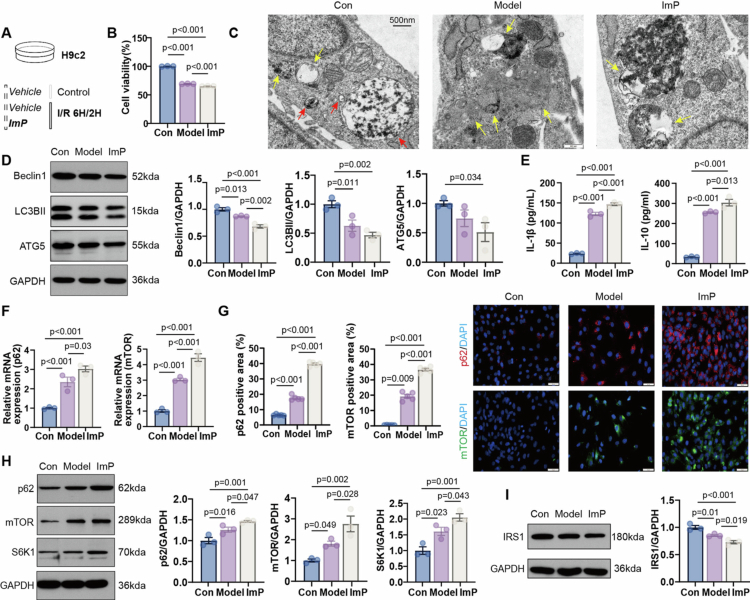
ImP aggravates H/R injury by impairing autophagy in cardiomyocytes. (A) Schematic of the *in vitro* experimental design. (B) H9c2 cell viability measured by CCK-8 (*n* = 3). (C) TEM of H9c2 cells showing autophagy-related structures (*n* = 3), red arrows indicate autophagosomes and yellow arrows indicate autolysosomes. (D) Beclin1, LC3B-II, and ATG5 protein levels in H9c2 cells (*n* = 3). (E) Levels of IL-1β and IL-10 in H9c2 cell supernatants (*n* = 3). (F) p62 and mTOR mRNA levels in H9c2 cells (*n* = 3). (G) Immunofluorescence staining of p62 (red) and mTOR (green) in H9c2 cells (*n* = 5). (H)WB analysis of p62, mTOR, S6K1 protein levels (*n* = 3). (I) WB analysis of IRS1 protein levels (*n* = 3).

We next tested whether ImP is sufficient to exacerbate H/R-induced cardiomyocyte injury and alter autophagy-related protein levels (Figure 4A). ImP reduced H9c2 viability in a dose-dependent manner (Figure S13). Increasing ImP concentrations were also associated with progressive alterations in autophagy-related protein expression in H9c2 cells (Figure S14). Using the selected ImP concentration, ImP further decreased cell viability after H/R (Figure 4B). Ultrastructural analysis by transmission electron microscopy showed fewer autophagosome structures, more severe mitochondrial damage, and an accumulation of mitochondrial fragments in ImP-treated cells (Figure 4C). Consistently, ImP decreased Beclin1, LC3B-II, and ATG5 abundance (Figure 4D), supporting an attenuation of autophagy-related processes in H/R-injured cardiomyocytes.

ImP directly altered inflammatory mediator output in H/R-injured H9c2 cells, as reflected by the changes in cytokine levels (Figure 4E). Alongside these inflammatory changes, H/R increased p62 and mTOR mRNA and protein abundance, and ImP further augmented both transcript and protein levels of the p62/mTOR axis (Figure 4F–H). In parallel, ImP increased the abundance of the downstream effector S6K1 (Figure 4H). Because p62/mTOR signaling has been linked to suppression of insulin signaling, and *F. nucleatum* exposure was associated with impaired insulin sensitivity (Figure S2), we assessed insulin signaling adapter IRS1. ImP was accompanied by reduced IRS1 abundance under H/R (Figure 4I). Together, these results indicate that ImP exacerbates H/R-induced cardiomyocyte injury and is accompanied by increased p62/mTOR abundance, altered S6K1 readouts, and reduced IRS1 abundance.

### ImP exacerbates MIRI with p62-dependent involvement of mTOR/S6K1 signaling

**Figure 5.**
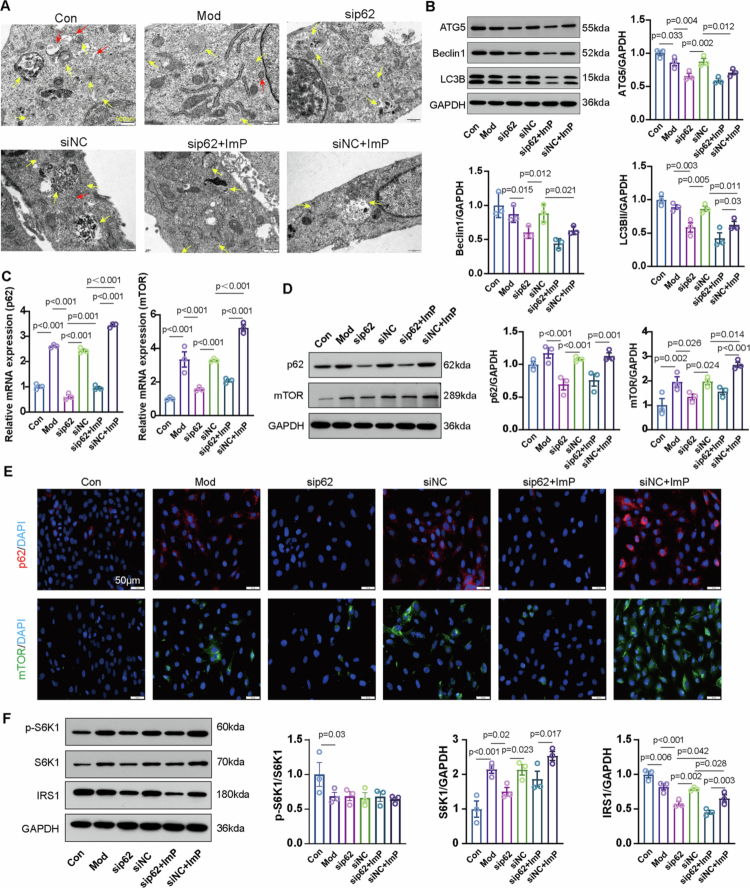
ImP activates mTOR/S6K1 in cardiomyocytes through p62. (A) Transmission electron microscopy (TEM) of H9c2 cells showing autophagy-related structures (*n* = 3); red arrows indicate autophagosomes and yellow arrows indicate autolysosomes. (B) Western blot (WB) analysis of ATG5, Beclin1, and LC3B-II in H9c2 cells (*n* = 3). (C) p62 and mTOR mRNA levels in H9c2 cells (*n* = 3). (D) WB analysis of p62 and mTOR protein levels in H9c2 cells (*n* = 3). (E) Immunofluorescence staining of p62 (red) and mTOR (green) in H9c2 cells (*n* = 5).(F) WB analysis of p-S6K1, S6K1, and IRS1 protein levels in H9c2 cells (n = 3).

To assess the requirement for p62 in ImP-associated autophagy-related alterations and IRS1 suppression, p62 was silenced in H9c2 cells. Under H/R + ImP, transmission electron microscopy revealed worsened mitochondrial damage with accumulation of autophagy-related vesicular structures; p62 silencing partially improved mitochondrial morphology and reduced this accumulation (Figure 5A). ImP reduced ATG5, Beclin1, and LC3B-II abundance under H/R (Figure 5B). p62 silencing also lowered these proteins and did not restore their levels in the presence of ImP (Figure 5B), indicating that these autophagy-related readouts are not rescued by limiting p62. In contrast, ImP markedly increased p62 and mTOR mRNA and protein abundance, which was substantially attenuated by p62 silencing (Figure 5C–E; Figure S15). Consistently, ImP altered total S6K1 abundance, whereas *p*-S6K1 was not significantly changed at this time point (Figure 5F). Inflammatory cytokines were elevated by ImP and were not significantly altered by p62 silencing (Figure S15). IRS1 abundance was reduced by ImP and remained suppressed despite p62 silencing (Figure 5F), suggesting that ImP-mediated IRS1 suppression is not fully p62 dependent.

**Figure 6.**
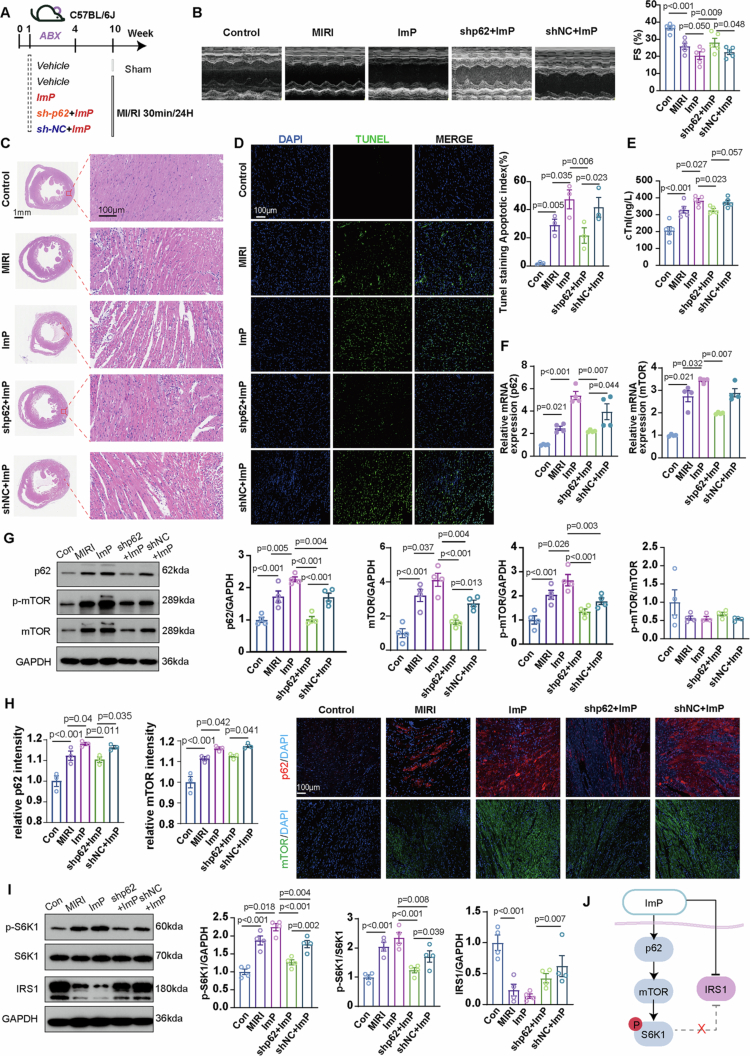
Cardiomyocyte-targeted p62 knockdown attenuates ImP-associated mTOR/S6K1 signaling *in vivo*. (A) Schematic of the *in vivo* study design. (B) Echocardiographic assessment of cardiac function and quantification of FS. (C) Representative H&E staining of heart sections. (D) Representative TUNEL staining of heart sections. (E) Plasma cTnI levels. (F) Cardiac p62 and mTOR mRNA levels. (G) Western blot analysis of *p*-p62, p62, *p*-mTOR, and mTOR. (H) Immunofluorescence staining of p62 (red) and mTOR (green). (I) Western blot analysis of *p*-S6K1, S6K1, and IRS1. (J) Proposed signaling model by which ImP modulates the p62/mTOR/S6K1 pathway in MIRI.

These findings were extended *in vivo* using cardiomyocyte-targeted AAV-shp62 combined with ImP administration and MIRI induction (Figure 6A). Relative to MIRI alone, ImP further reduced fractional shortening and increased myocardial necrosis and TUNEL positivity, whereas AAV-shp62 improved cardiac function in the MIRI + ImP setting and attenuated injury phenotypes, accompanied by lower plasma cTnI and reduced apoptosis-related markers (Figure 6B–E and Figure S16). ImP increased cardiac p62 and mTOR mRNA and protein abundance (Figure 6F–H) and elevated *p*-S6K1 (Figure 6I); these effects were suppressed by AAV-shp62 (Figure 6F–I), supporting a p62-dependent contribution to mTOR/S6K1 signaling *in vivo*. Notably, IRS1 remained reduced in the MIRI + ImP setting and was not restored by p62 knockdown (Figure 6I). Together, these findings support a p62-sensitive mTOR/S6K1 signaling branch in ImP-associated injury, whereas IRS1 suppression was not rescued by p62 knockdown, suggesting that additional mechanisms may contribute to impaired insulin signaling (Figure 6J).

## Discussion

This study supports an oral–gut–heart pathway linking an oral pathobiont to myocardial ischemia–reperfusion injury. The principal finding is that oral *F. nucleatum* worsened MIRI in association with gut microbial functional remodeling and increased circulating imidazole propionate (ImP), supporting a mechanism whereby oral dysbiosis influences distal cardiac injury through altered gut microbial metabolism rather than through distal bacterial burden alone. This framework expands the cardiovascular relevance of oral dysbiosis from direct bacterial pathogenicity toward cross-compartment functional interactions^19^^,^^23^^,^^32^ that generate circulating mediators capable of amplifying cardiac injury.^33^

An important question is how an oral pathobiont such as *F. nucleatum* may influence the gut microbiota without persistent distal expansion. Current evidence indicates that oral–gut microbial crosstalk does not necessarily require stable long-term intestinal engraftment.^34^ Studies suggest that oral bacteria are subject to substantial ecological filtering in the distal gut, implying that their systemic effects may result less from sustained colonization by the original oral organism than from community-level remodeling of gut microbiota.^35^^,^^36^ In parallel, oral microbiota may also modulate intestinal immune tone,^37^ thereby reshaping the ecological niche in which gut metabolic functions are executed.^35^ In addition, the oral–gut axis also involves virome interactions, particularly bacteriophages.^38^^,^^39^ Our data raise the possibility that oral *F. nucleatum* may remodel the gut microbial ecosystem, potentially through altered substrate availability,^25^ interspecies cross-feeding,^40^^,^^41^ and local redox conditions,^42^^,^^43^ thereby shifting microbial histidine-metabolic capacity and favoring a gut configuration permissive for ImP production.^29^^,^^44^ Consistent with this model, ImP^26‐29,45,46^ appears to represent a metabolite-level output of the oral–gut interaction identified in this study. Plasma ImP was elevated in patients with CHD, supporting the clinical relevance of this metabolite signal. Our metagenomic analyzes further support a two-step model in which *F. nucleatum* is linked to the histidine-to-urocanate step through HAL-associated function, whereas the terminal conversion from urocanate to ImP depends on UAR-associated gut microbial capacity.

In cardiomyocytes subjected to H/R, ImP increased p62 and mTOR mRNA and protein abundance and altered S6K1 expression, concomitant with aggravated injury. Autophagy-related molecular changes did not support uniform autophagy activation; instead, ImP reduced several core autophagy-related proteins, including ATG5, Beclin1, and LC3B-II,^47^ and was accompanied by autophagy-associated ultrastructural abnormalities and mitochondrial damage.^48^ These findings suggest that ImP disrupts autophagy homeostasis under stress conditions. Among the signaling alterations observed, the p62/mTOR/S6K1 axis emerged as a major ImP-responsive pathway, as p62 silencing *in vitro* and cardiomyocyte-targeted p62 knockdown *in vivo* attenuated ImP-associated signaling activation and mitigated myocardial injury. These data support involvement of p62-dependent mTOR/S6K1 signaling in mediating the deleterious cardiac effects of ImP.^49^^,^^50^ S6K1-related signaling changes differed between *in vitro* and *in vivo* settings, which may reflect the marked temporal and context dependence of S6K1 phosphorylation, as well as differences in sampling windows and systemic influences present *in vivo*.^51^^,^^52^

IRS1 suppression persisted despite p62 knockdown, indicating that ImP also acts through mechanisms not fully dependent on p62.^53^^,^^54^ This finding suggests that the cardiac effects of ImP are not restricted to a single signaling branch.^55^ One possible explanation is that, in addition to activating p62-dependent mTOR/S6K1 signaling, ImP may impair insulin-related signaling through parallel mechanisms involving stress kinase activation, oxidative stress, inflammatory signaling, or other nutrient-sensing pathways.^56^

Several limitations should be acknowledged. First, although the integrated multiomics, microbiota depletion, metabolite intervention, and gene-silencing data collectively support the proposed oral–gut–heart pathway, direct causal testing of the gut microbial component remains incomplete. In particular, the present study does not establish whether the remodeled gut microbiota is sufficient to transfer the aggravated phenotype, nor does it define which UAR-positive bacteria are required for ImP elevation *in vivo*. These will require further investigation using fecal microbiota transplantation, bacterial tracking, defined coculture or colonization systems, and direct manipulation of microbial UAR activity. Second, the mechanistic cell experiments established biological activity and enabled focused pathway interrogation; broader analyzes will be necessary to define more precisely the quantitative relationship between ImP exposure, autophagy dysregulation, IRS1 pathway, and myocardial injury.

In summary, the present work suggests an oral–gut axis mechanism in which an oral pathobiont reshapes gut microbial function to elevate a circulating metabolite that amplifies MIRI, providing a conceptually and mechanistically grounded extension of oral dysbiosis-associated cardiovascular risk.

## Conclusions

Our study supports the existence of an oral–gut axis in which *F. nucleatum* aggravates MIRI through remodeling gut microbial metabolic output. *F. nucleatum* was associated with increased gut *Lactobacillus* abundance and elevated circulating ImP, a gut microbiota-derived metabolite that is also elevated in patients with CHD. Microbiota depletion lowered circulating ImP and attenuated MIRI, whereas ImP aggravated injury and engaged a p62-sensitive mTOR/S6K1 signaling branch in the heart, accompanied by autophagy-related molecular and ultrastructural alterations; in parallel, IRS1 suppression persisted despite p62 knockdown. Together, these findings highlight cross-compartment microbial functional interactions as an underappreciated mechanism linking oral dysbiosis to reperfusion injury risk and support microbiota-derived ImP and oral–gut microbial metabolic remodeling as potential targets for MIRI prevention and intervention.

## Supplementary Material

supplementary Material supplementary_fig0421

supplementary TableS.xlsx supplementary TableS.xlsx

## Data Availability

Publicly available metagenomic sequencing data are available in the Genome Sequence Archive under accession number PRJCA024368. All other data supporting the findings of this study are available within the article and its supplementary materials.
