# Supplementary material for: An oral–gut microbial metabolite links Fusobacterium nucleatum to aggravated myocardial ischemia–reperfusion injury
Source: Gut Microbes. 2026 Apr 25;18(1):2662082. doi: 10.1080/19490976.2026.2662082 (PMC13114118; doi:10.1080/19490976.2026.2662082)
Supplement: supplementary Material — supplementary_fig0421 [file KGMI_A_2662082_SM1958.docx]

**Supporting Information**

**An oral–gut microbial metabolite links *Fusobacterium nucleatum* to aggravated myocardial ischemia–reperfusion injury**

Yiwen Li, Qian Xu, Mengmeng Zhu, Wenting Wang, Yanfei Liu, Hongjun Yang*, Yue Liu*

E-mail: liuyueheart@hotmail.com (Y.L., Yue Liu); hongjun0420@vip.sina.com (H.Y., Hongjun Yang).

**This file includes:**

Figs. S1 to S16


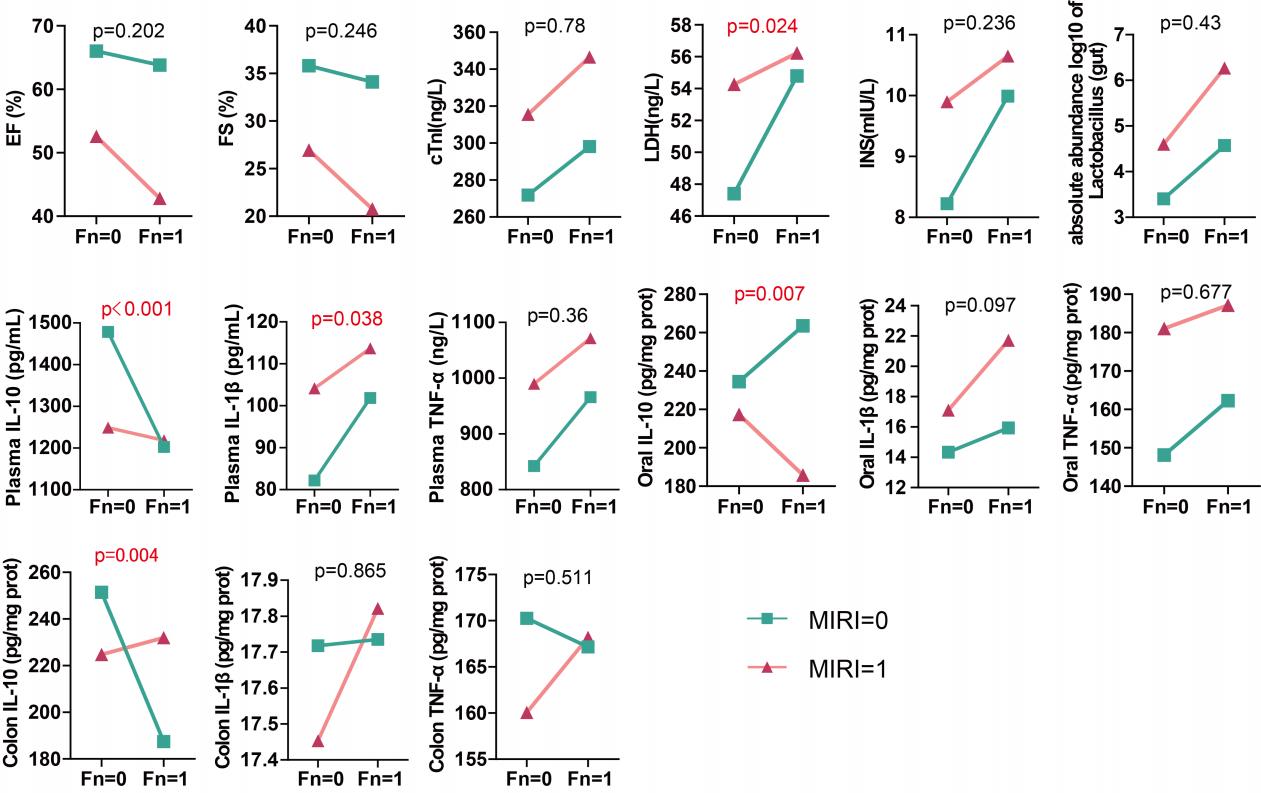


**Fig. S1. Interaction analysis between oral *F. nucleatum* exposure and MIRI.** Two-way interaction effects with bacterial treatment (vehicle vs. *F. nucleatum*) and surgical condition (sham vs. MIRI) as fixed factors. Echocardiographic indices (EF and FS), plasma injury markers (cTnI and LDH), insulin (INS), gut *Lactobacillus* abundance, and inflammatory cytokines (IL-1β, TNF-α, and IL-10) measured in plasma, oral samples, and colonic tissue were evaluated; where interaction was significant, post hoc comparisons were performed between vehicle and *F. nucleatum* within each surgical condition. Fn = 0 indicates PBS gavage and Fn = 1 indicates *F. nucleatum* gavage; MIRI = 0 indicates sham surgery and MIRI = 1 indicates ischemia–reperfusion (MIRI).


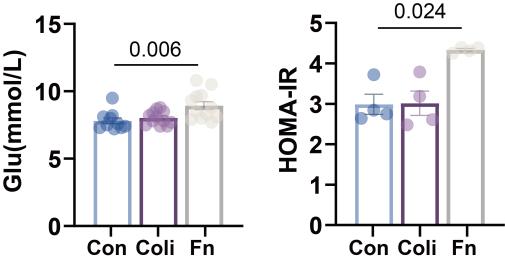


**Fig. S2. Oral F.n promotes insulin resistance.** Blood glucose levels (n = 10–12) and the homeostasis model assessment of insulin resistance (HOMA-IR; n = 4) were measured in mice receiving Control, *E.coli,* or F.n gavage; oral F.n increased insulin resistance relative to controls.


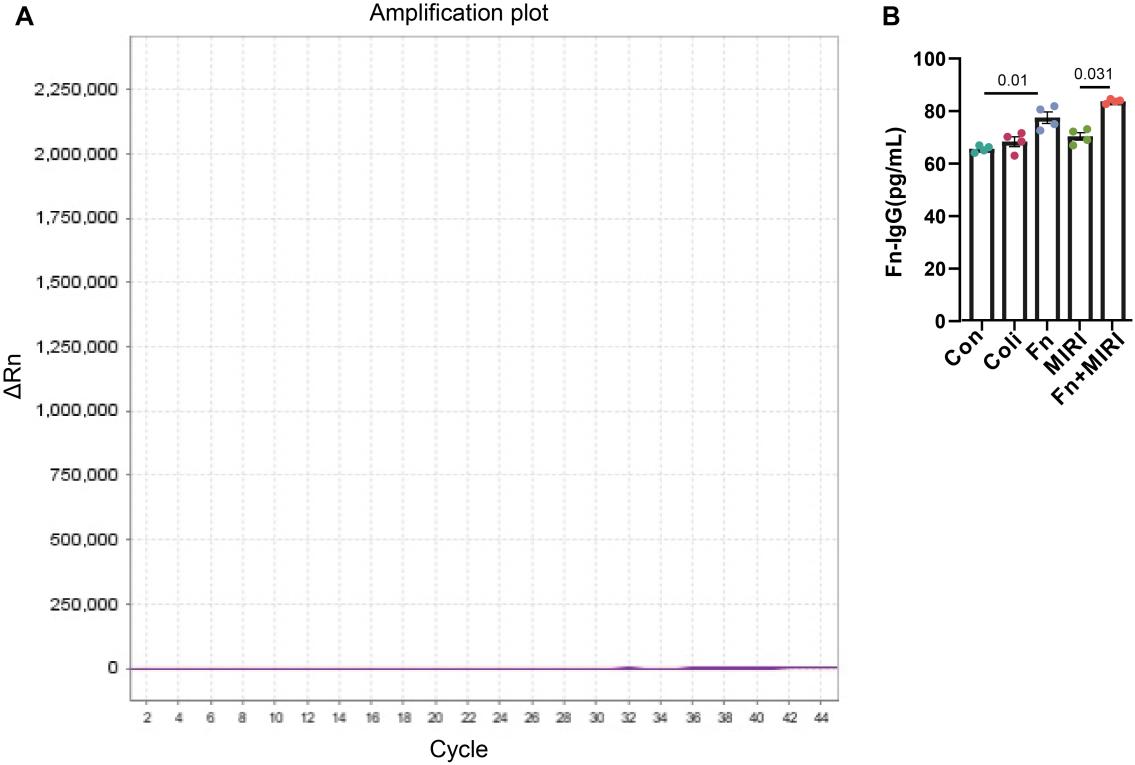


**Fig. S3. Oral F.n does not establish detectable gut colonization but elicits host responses.** (A) Melting-curve analysis of F.n–specific PCR in gut samples. (B) Plasma anti–F.n IgG levels in mice from each group (n = 4).


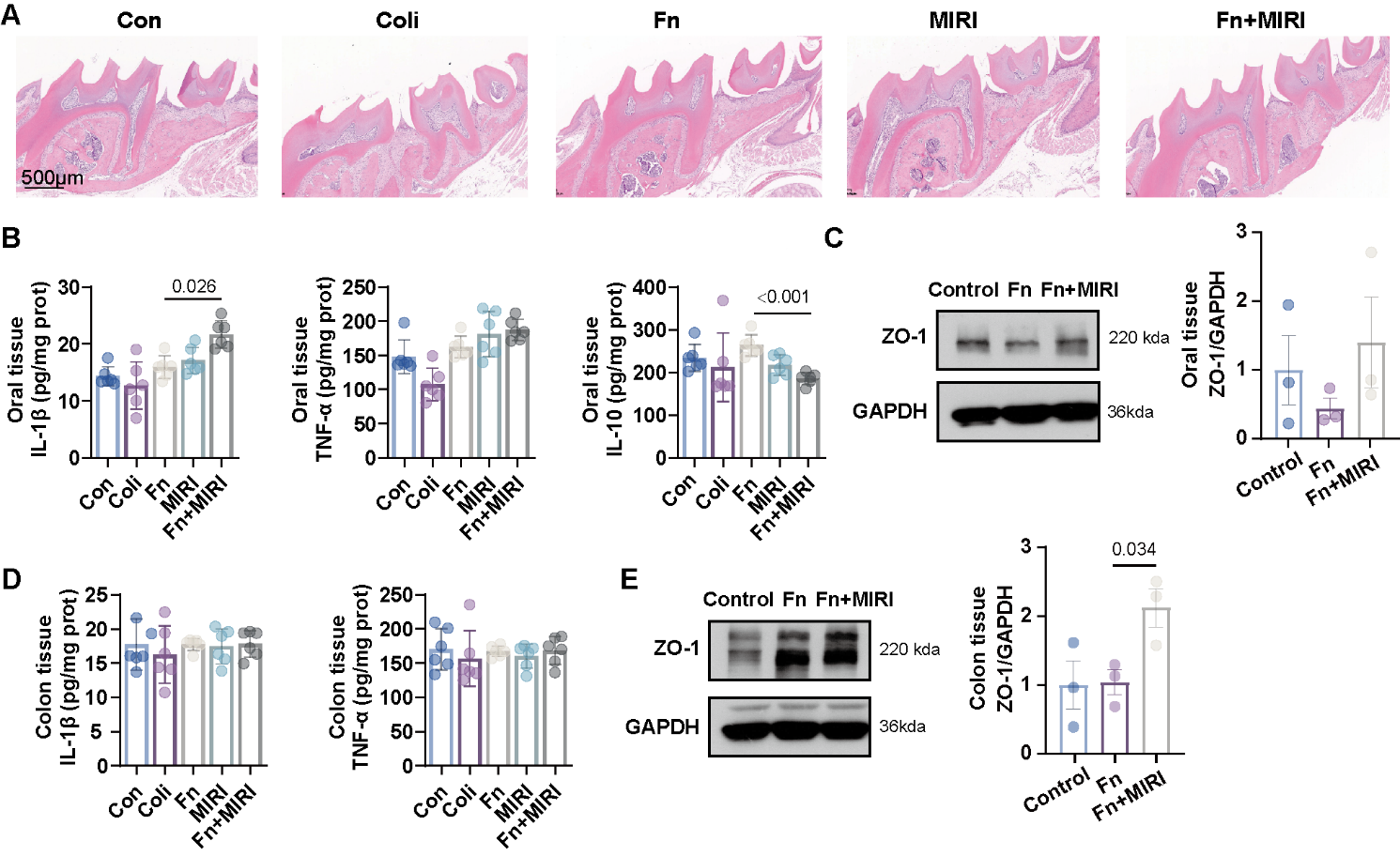


**Fig. S4. F.n exacerbates MIRI without overt gut barrier disruption.** (A) Representative H&E staining of oral tissues from each group (n = 3). (B) Levels of IL-1β, TNF-α, and IL-10 in oral tissues (n = 6). (C) Representative Western blot bands and quantitative analysis results for the tight junction-associated protein ZO-1 in mouse oral tissue (n=3).(D)Colonic levels of IL-1 and TNF-α (n = 6). (E) Representative Western blot bands and quantitative analysis results for the tight junction-associated protein ZO-1 in mouse colon tissue (n=3).


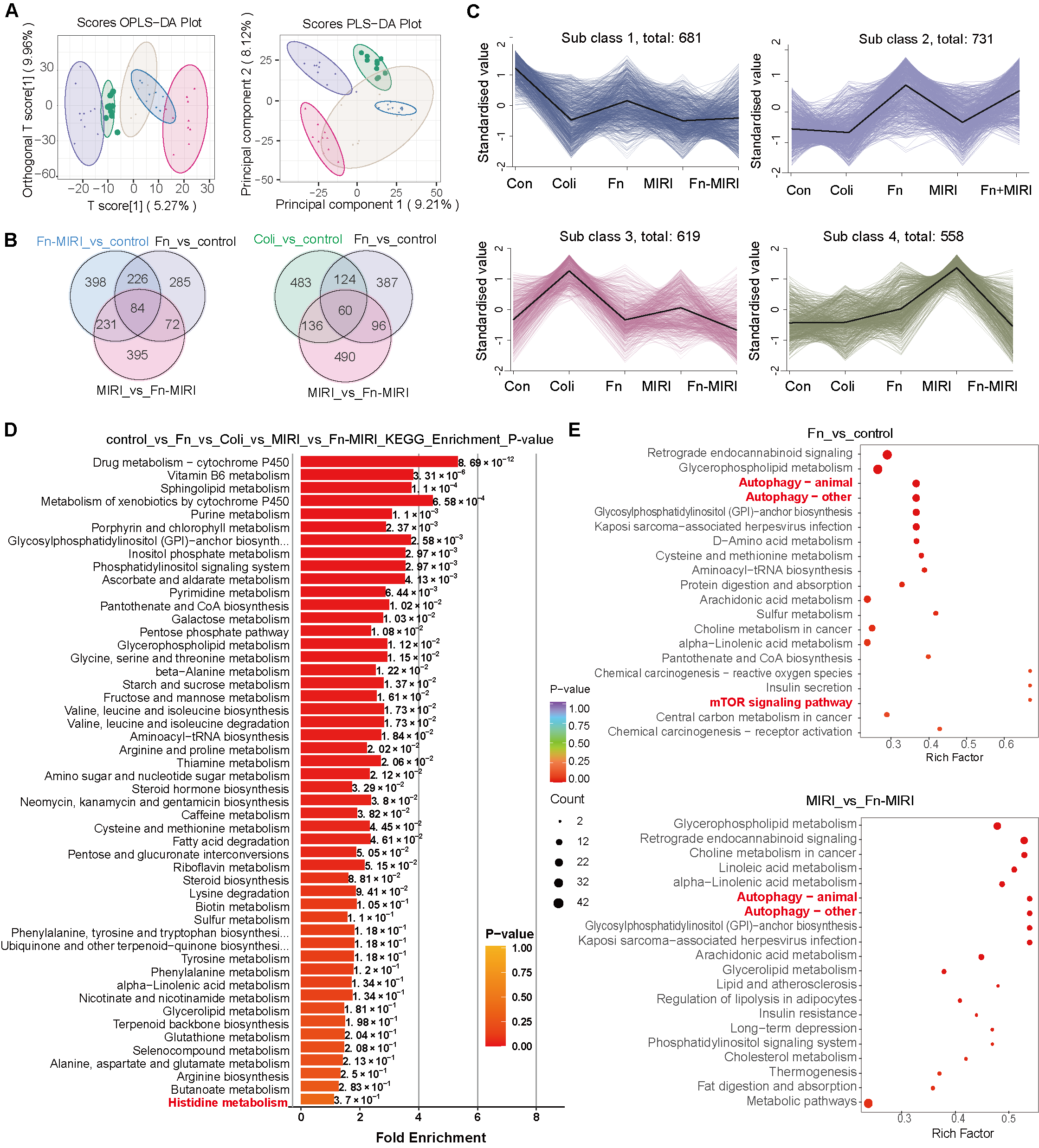


**Fig. S5. Additional metabolomics analyses.** (A) OPLS-DA and PLS-DA score plots of metabolomic profiles across groups. (B) Venn diagram of differentially abundant metabolites. (C) k-means clustering identifying four metabolite subclusters with distinct abundance trajectories. (D) KEGG pathway enrichment analysis of differential metabolites between groups. (E) Bubble plots showing significantly enriched pathways in the comparisons of F.n vs Control and MIRI vs F.n–MIRI.


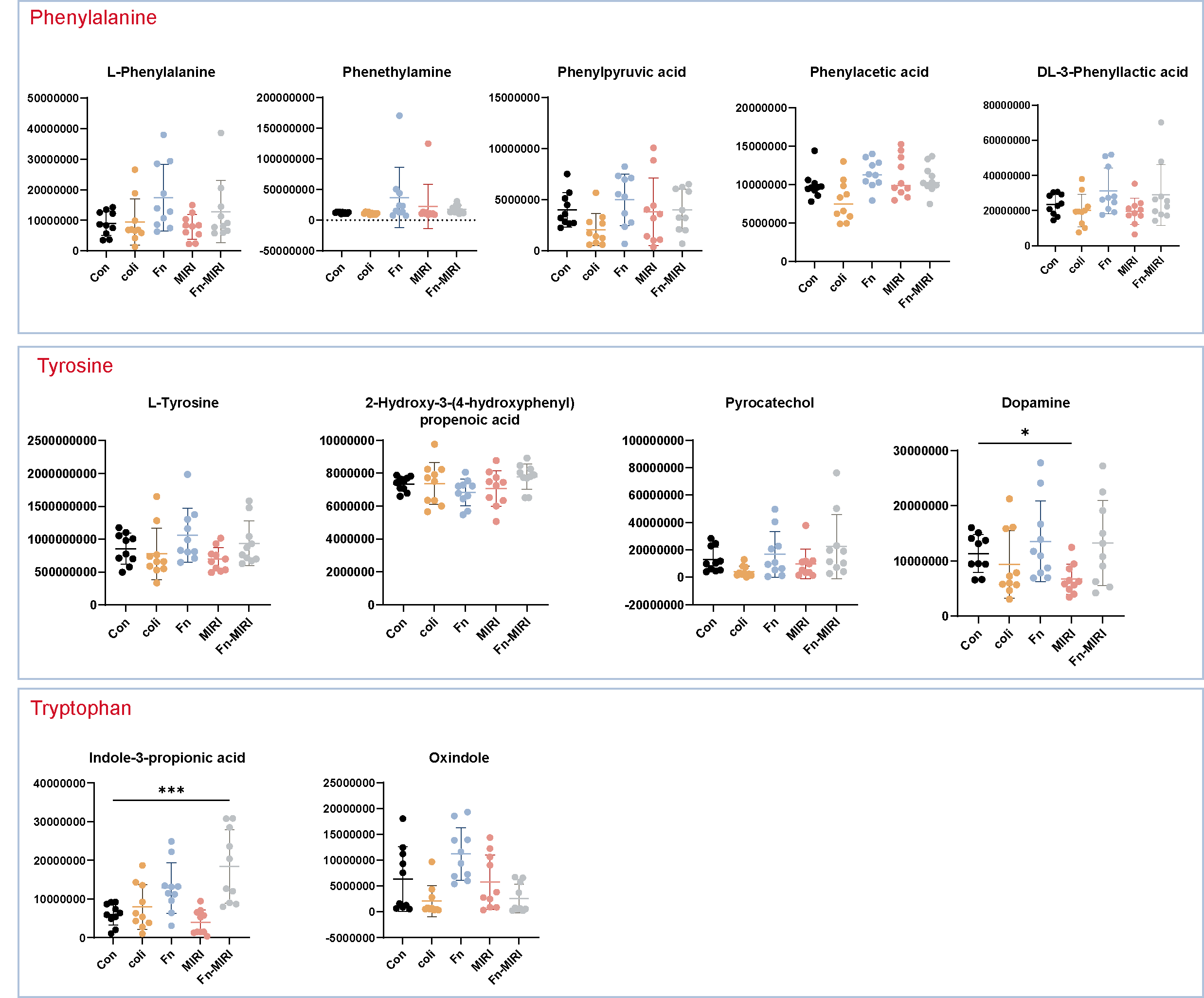


**Fig. S6. Comparison of aromatic amino acid metabolites identified in Cluster 2 by untargeted metabolomics.****P*＜0.05，***P*＜0.01，****P*＜0.001.


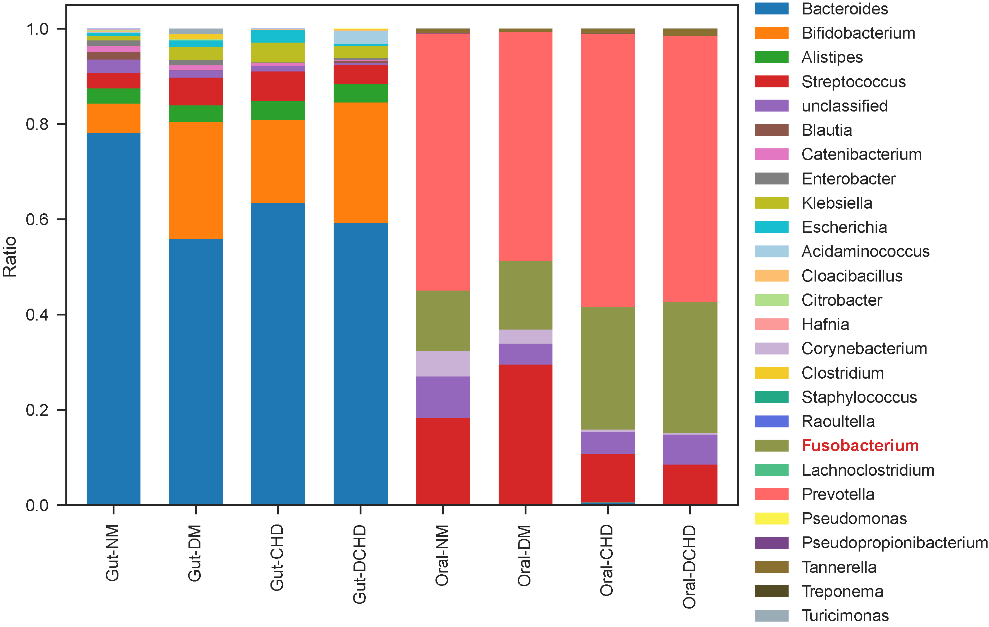


**Fig. S7.** **Genus-level composition of HAL-encoding bacteria in the oral and gut microbiomes across study groups.**


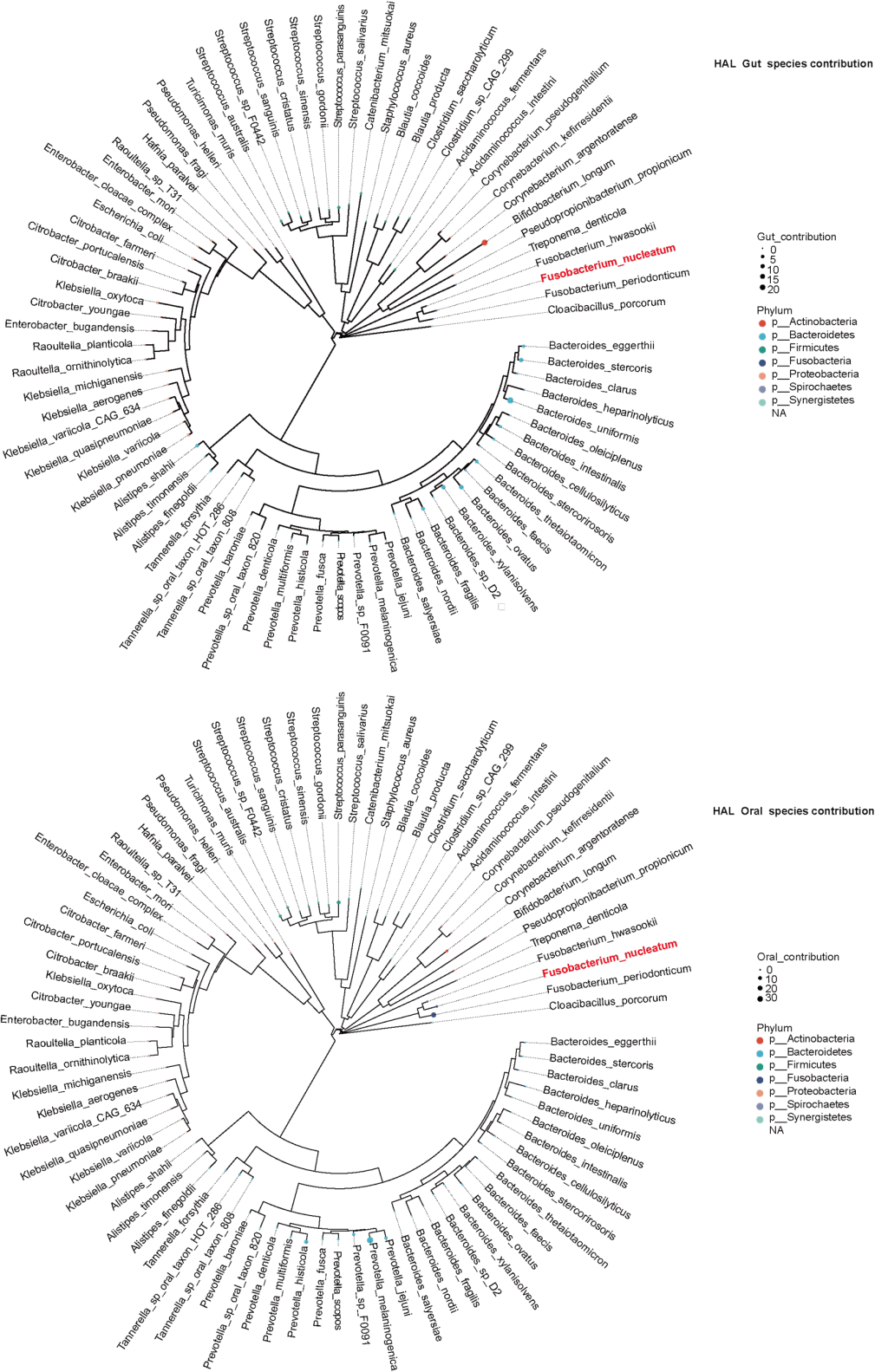


**Fig. S8. Phylogenetic tree of HAL–encoding taxa in the oral and gut microbiomes.**


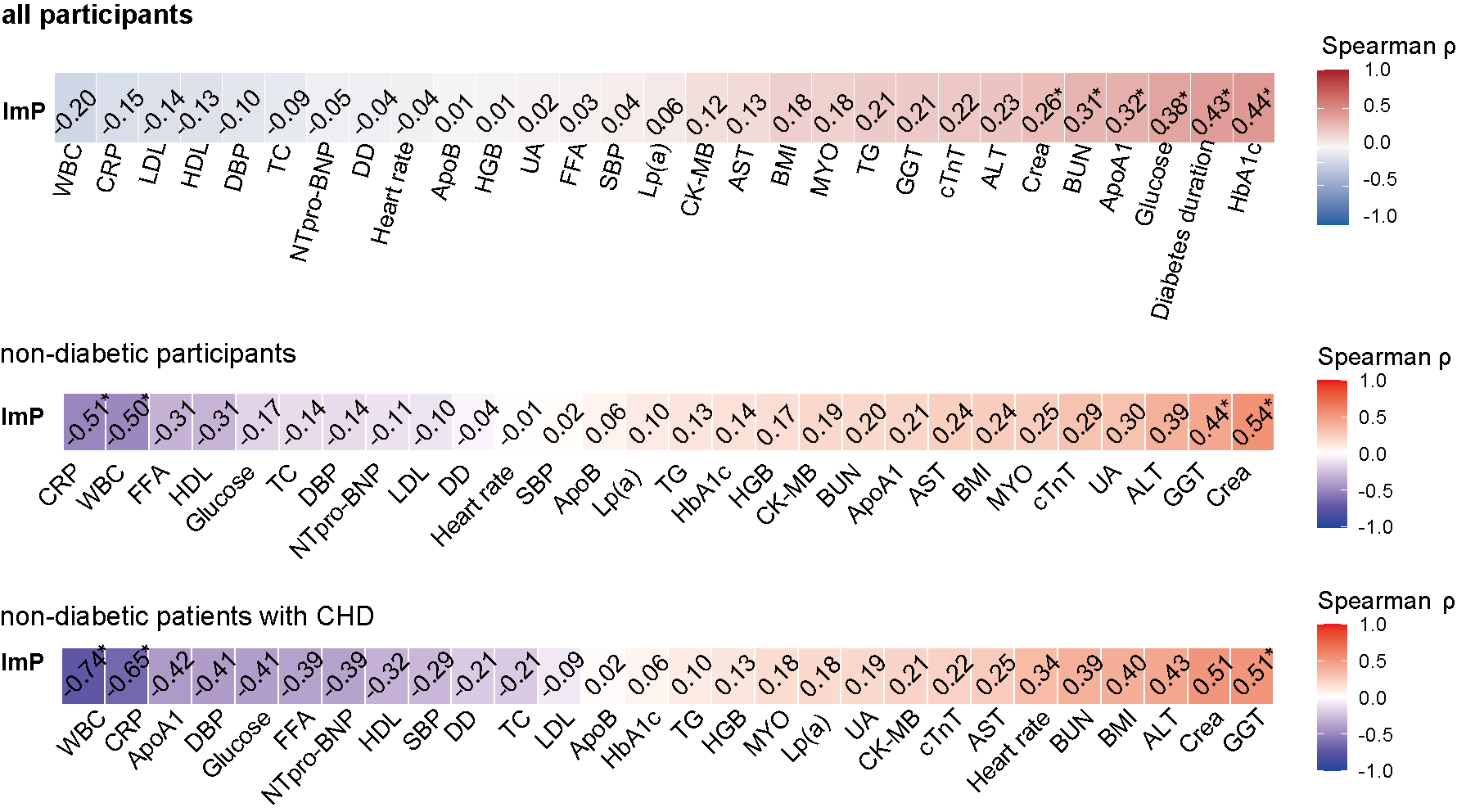


**Fig. S9. Correlation between plasma ImP and clinical parameters in participants.**


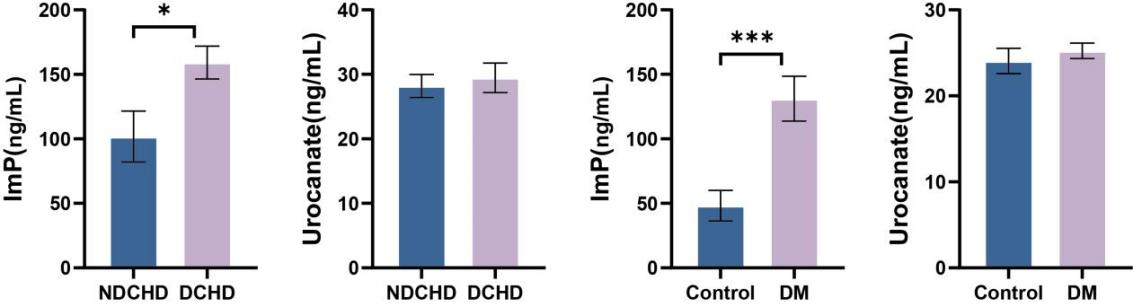


**Fig. S10. Stratified analysis of urocanate and ImP by diabetes status.** Within the CHD cohort, participants were stratified by diabetes status; plasma ImP levels were significantly higher in the diabetic CHD group (DCHD, n = 16) than in the nondiabetic CHD group (NDCHD, n = 15), whereas urocanate levels did not differ significantly between groups. In the MMC cohort, plasma ImP levels were also significantly higher in participants with diabetes (DM, n = 17) than in those without diabetes (Control, n = 15), with no significant difference in urocanate levels. **P* < 0.05, ***P* < 0.01, ****P* < 0.001.


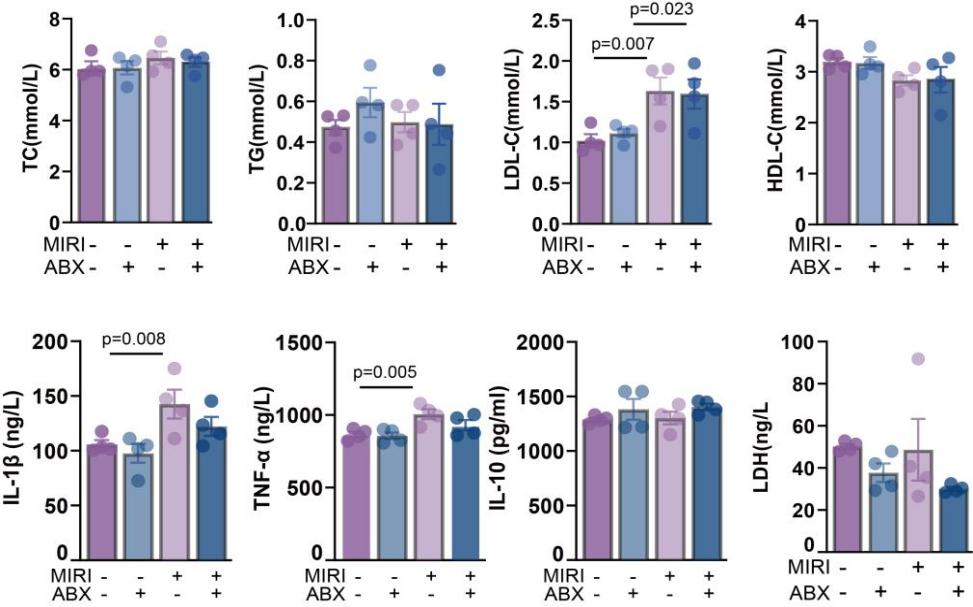


**Fig. S11. ABX experiment: plasma lipid metabolic indices and inflammatory cytokines.** Plasma lipid metabolism–related parameters and circulating inflammatory cytokines were measured across groups in the ABX mouse experiment (n = 4).


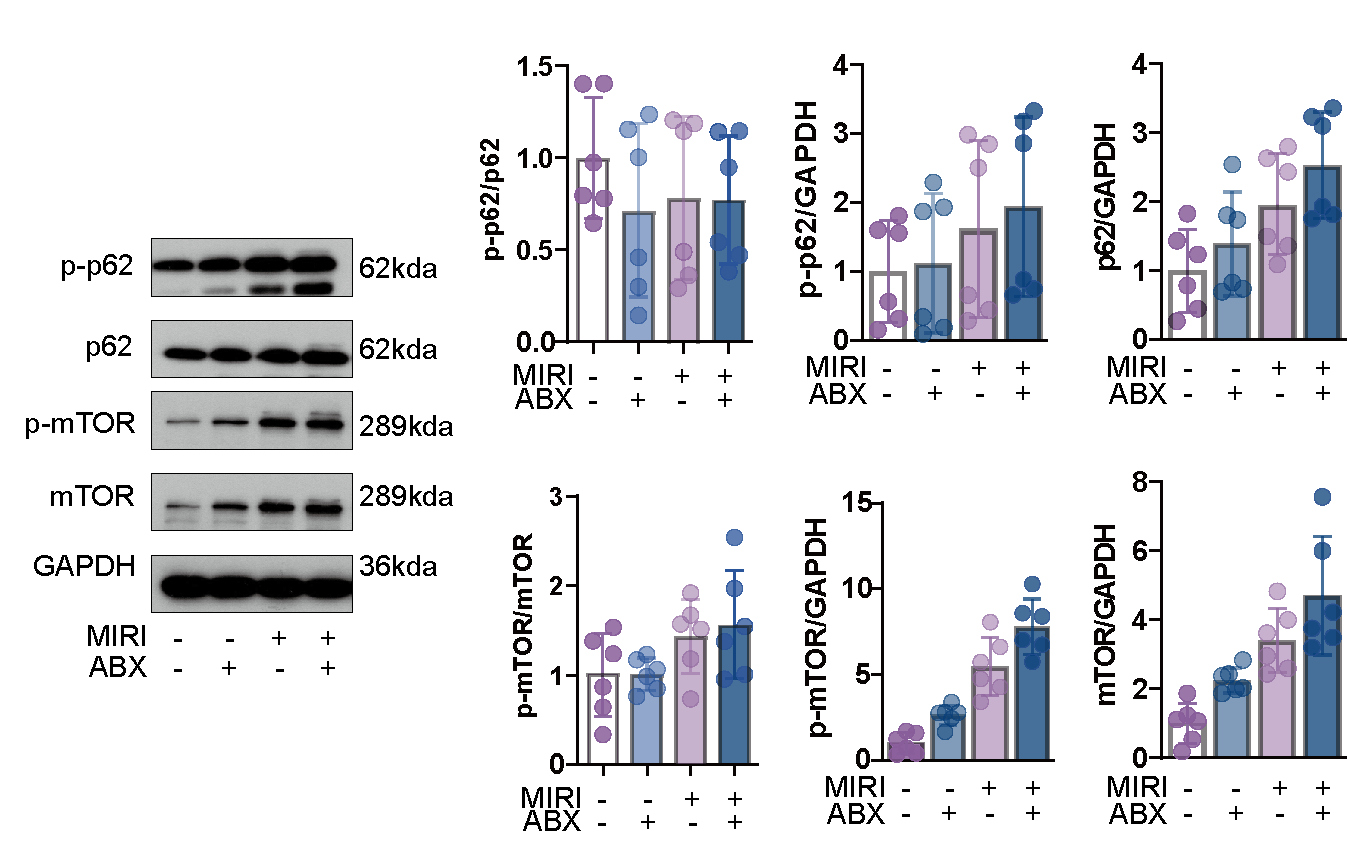


**Fig. S12. ABX experiment: quantification of cardiac p62/mTOR signaling and phosphorylation readouts.**Densitometric quantification of immunoblot signals for cardiac p62, mTOR, and their phosphorylated forms in the ABX mouse experiment (n = 6).


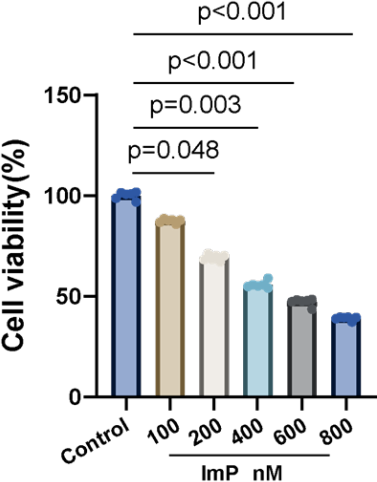


**Fig. S13. ImP dose–response in vitro: H9c2 cell viability.** CCK-8 assay of H9c2 cell viability following treatment with a concentration gradient of ImP(n = 6).


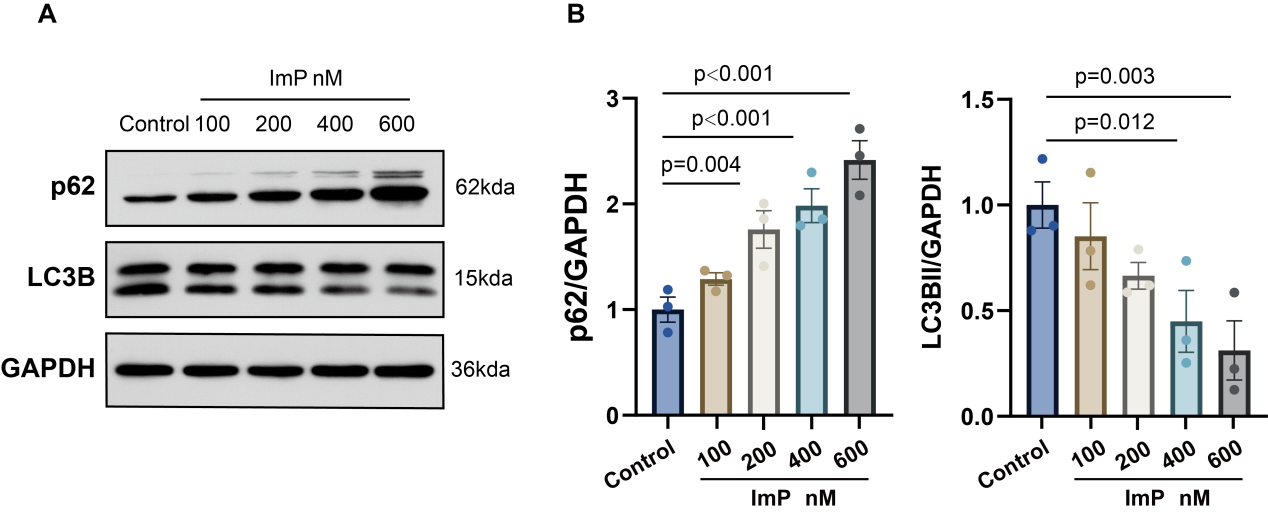


**Fig. S14. ImP dose–response in vitro: expression of autophagy-related proteins.** (A) Representative Western blot bands for the autophagy-related proteins p62 and LC3B-II following treatment of H9c2 cardiomyocytes with different concentrations of ImP (0-600 nM); (B) Quantitative analysis of the grayscale values ​​of the p62 and LC3B-II protein bands (n=3).


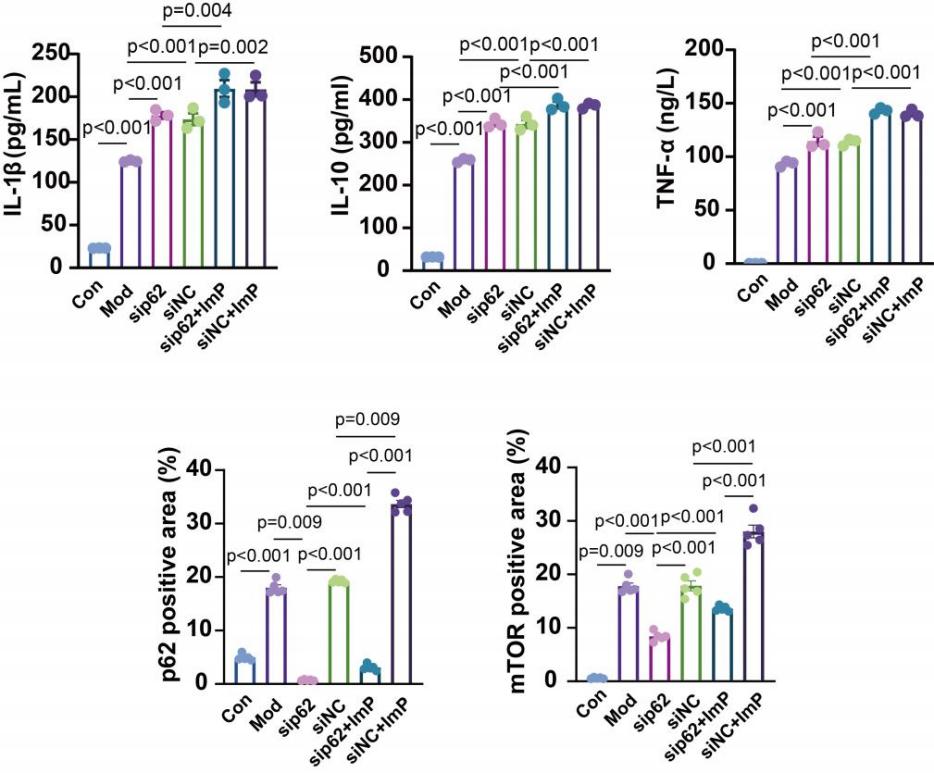


**Fig. S15. In vitro p62 silencing plus ImP: inflammatory cytokines and p62/mTOR immunofluorescence quantification.** Levels of IL-1β, IL-10, and TNF-α in H9c2 cells across treatment groups (n = 3), and quantitative analysis of p62 and mTOR immunofluorescence signals (n = 5).


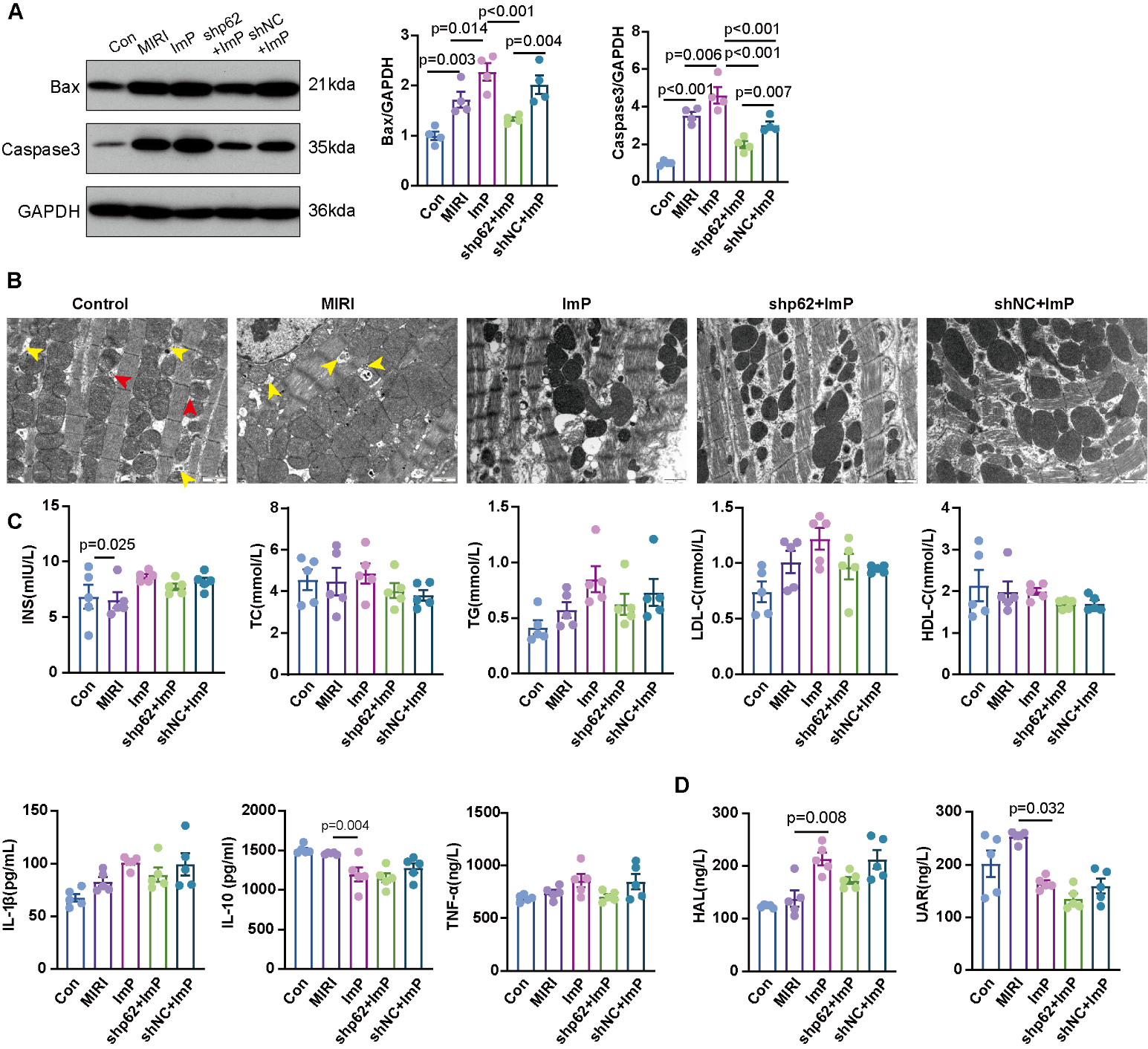


**Fig. S16. In vivo p62 silencing attenuates ImP-associated injury readouts and alters autophagy.**(A) Immunoblot analysis and densitometric quantification of apoptosis- related proteins (Bax and caspase-3; normalized to GAPDH) in mouse hearts across groups (Control, MIRI, ImP, shp62+ImP, and shNC+ImP; n = 4). (B) Representative transmission electron microscopy images of myocardium (n = 3); red arrowheads indicate autophagosomes and yellow arrowheads indicate autolysosomes. (C) ELISA measurements in plasma(n = 5) for insulin (INS), lipid indices (TC, TG, LDL-C, and HDL-C), inflammatory cytokines (IL-1β, IL-10, and TNF-α).(D) histidine–ImP pathway enzymes (HAL and UAR n=5).
